# Primiparous women differ from multiparous women after early discharge regarding breastfeeding, anxiety, and insecurity: A prospective cohort study

**DOI:** 10.18332/ejm/146897

**Published:** 2022-03-10

**Authors:** Victoria Lindblad, Dorte Melgaard, Kristine L. Jensen, Anya Eidhammer, Signe Westmark, Kristian H. Kragholm, Ditte Gommesen

**Affiliations:** 1 Department of Gynecology and Obstetrics, North Denmark Regional Hospital, Hjørring, Denmark; 2 Center for Clinical Research, North Denmark Regional Hospital, Hjørring, Denmark; 3 Department of Clinical Medicine and Clinical Research, Aalborg University, Aalborg, Denmark; 4 Unit of Clinical Biostatistics and Epidemiology, Aalborg University Hospital, Aalborg, Denmark; 5 Department of Clinical Research, Faculty of Health Science, University of Southern Denmark, Odense, Denmark

**Keywords:** primiparity, multiparity, length of stay, postnatal care, infant, early discharge

## Abstract

**INTRODUCTION:**

Breastfeeding and factors influencing breastfeeding are essential when considering the association between parity and neonatal and maternal morbidity risks when mothers are discharged within 24 hours after birth. However, there is a lack of studies examining the effect of parity and breastfeeding in a setting where all healthy mothers are recommended discharge four hours after birth. Therefore, this study examined the association between parity and the time for discharge, breastfeeding, and factors influencing breastfeeding.

**METHODS:**

The study was designed as a prospective cohort study. Data were obtained from questionnaires at one and at six weeks after birth, and combined with registered data. All 147 included mothers were healthy, with an uncomplicated birth and a healthy newborn, discharged within 24 hours after birth.

**RESULTS:**

This study documented that primiparous women had a higher relative risk (RR=2.62; 95% CI: 1.35–5.10) of having doubts about infant feeding after discharge than multiparous women. Furthermore, 54% of primiparous women contacted the maternity ward after discharge compared to 27% of multiparous women. Twice as many primiparous than multiparous women felt anxious or depressed at one and at six weeks after birth. Finally, the study documented that 13% of primiparous women and 5% of multiparous women discharged within six hours after birth perceived the time before discharge to be too short.

**CONCLUSIONS:**

Primiparous women differ from multiparous women regarding breastfeeding, insecurity, and anxiety. Special attention towards primiparous women and a follow-up strategy that allows the mothers to contact the maternity ward after early discharge is recommended.

## INTRODUCTION

Studies since 2016 from developed and developing countries document that early discharge after birth is becoming more common among mothers^1-3^. Thus, studies found that up to 28% of primiparous women left the hospital within 24 hours after birth^1-3^. In comparison, up to 84% of multiparous women were discharged within 24 hours after birth^1-3^. However, concerns have been raised regarding early discharge and an increase in neonatal and maternal morbidity^1,4^. Healthcare professionals recommend breastfeeding to prevent neonatal infections and protect against overweight and diabetes later in life^5,6^. Furthermore, breastfeeding might reduce the mothers’ risk of diabetes, breast cancer, and ovarian cancer^5^. However, problems with breastfeeding in the first week after birth increases the risk of neonatal jaundice, dehydration, weight loss, and readmission^6^. Symptoms of neonatal morbidity due to problems with breastfeeding usually do not occur until after the first day in the newborn’s life^6-9^. Thus, paying attention to detailed information on morbidity symptoms to the parents may be crucial. A study from the US in 2015 found that 35% of primiparous women had problems with breastfeeding during a 48 hours hospital stay after birth compared to 20% of multiparous women^10^. Another study observed no difference in breastfeeding incidence, six to eight weeks after birth, in two groups of primiparous women leaving the hospital 24 and 48 hours after birth, respectively^11^. Further, mothers feeling anxious or depressed after birth are at greater risk for discontinuing breastfeeding^11,12^, and primiparous women have been found to have a higher postpartum anxiety and depression rate than multiparous women^13^. However, another study observed no difference in depression incidence in women discharged before and after 72 hours after birth^14^. In addition, mothers’ insecurity and readiness for discharge might influence breastfeeding^11,14^. Therefore, breastfeeding and factors influencing breastfeeding are essential when considering the association between parity and neonatal and maternal morbidity risks when mothers are discharged within 24 hours. Two systematic reviews compared early discharge with the standard length of hospital stay after birth and found that it was safe for healthy mothers and term infants to be discharged early after birth if accompanied by one home visit by a midwife^4,15^. However, the definition of early discharge in the reviews varied from six to 72 hours after birth. Therefore, this study examined the association between parity and the time for discharge, breastfeeding, and the factors influencing breastfeeding among mothers discharged within 24 hours after birth.

## METHODS

This article follows the checklist according to the ‘Strengthening The Reporting of Observational Studies in Epidemiology’ (STROBE) statement^16^. The study was a prospective cohort study among women giving birth vaginally in an urban hospital and being discharged within 24 hours after birth. Participants were recruited between 20 September 2020 and 16 April 2021 from the Department of Gynecology and Obstetrics at North Denmark Regional Hospital, with has approximately 1400 births annually. A midwife invited the mothers to participate in this study at a routine biochemical screening of the newborns 48 to 72 hours after birth. Inclusion criteria were: mothers with vaginal birth, gestational age of 37+0 to 42+6 weeks, reading and understanding Danish, and discharged within 24 hours. Exclusion criteria were: caesarean section, stillbirth, and homebirth. The standard recommendation was a discharge of all healthy mothers, with an uncomplicated vaginal birth and healthy newborns, four hours after birth. See the labor wards criteria and contraindications for discharge four hours after birth in Supplementary file Table S1. All mothers received information about the mothers’ vaginal bleeding, fever, and signs of neonatal jaundice before discharge. Furthermore, all mothers were handed out a schedule of how often the newborn needs to urinate, breastfeed and how the feces change color day-by-day within the first week after birth. The labor wards follow-up strategy is presented in Supplementary file Table S2. We define early discharge as discharge within 24 hours after birth.

A self-administrated online questionnaire was used to collect data and contained 44 questions. Seven questions from the questionnaire were used for this study (see questions and answer possibilities in the study in Supplementary file Table S3)^17,18^. A link to the questionnaire was sent to the mother’s security mail one week after birth, and by e-mail, six weeks after birth.

If the mother did not respond to the questionnaire, she was contacted by phone 10 to 11 days after birth and encouraged to fill in the questionnaire. A reminder was sent by phone if there was a lack of contact after two calls. If the mother was contacted by phone with no response after four days, another reminder was sent by phone. Baseline characteristics were retrieved from the regions local department of data management. The software program Research Electronic Data Capture (REDCap^®^) hosted by the Region of Northern Denmark was used to manage the questionnaires^19,20^. REDCap is a secure web-based software platform designed to support data capture for research studies. Data from the local register were combined with the questionnaire using the unique personal identification number given at birth to all Danish individuals by the Danish Civil Registration system^21^.

The independent variable was parity. The primary outcome was breastfeeding at one and at six weeks after birth. The secondary outcomes were neonatal readmission within seven days after discharge and mothers who felt little, much, or extremely anxious or depressed versus not feeling anxious or depressed at one and at six weeks after birth. Furthermore, secondary outcomes were mothers who perceived the length of hospital stay after birth as too short versus appropriate or too long, and mothers who contacted the maternity ward within seven days after discharge. Lastly, secondary outcomes were mothers who thought they had little or much knowledge of the newborn’s well-being versus not knowing any signs of the newborn’s well-being and mothers who had doubts about infant feeding after discharge. Multiparous women were set as the reference group. Expected early discharge is defined as discharge within six hours after birth from the labor ward. Regarding the mothers’ perception of the length of hospital stay after discharge, we divided the mothers into mothers discharged within 24 hours after birth and discharged within six hours, and between seven to 24 hours.

### Statistical analysis

We performed a sample size calculation based on the available literature with a significance level of 0.05 and a power of 80. Our sample size calculation based on the literature found that a minimum of 384 mothers were required to detect a difference between parity and breastfeeding^10^. Categorical variables were presented as numbers and proportions. Normal distributed continuous variables were presented as means with standard deviation. Continuous variables not normally distributed were presented as median and range between 1st and 3rd quantile. We visually evaluated the normal distribution of the continuous data by inspecting histograms and quantile-quantile plots. Fischer’s exact test was used to test the statistical difference between categorical variables and parity. Student’s t-test was used to test the statistical difference between parity groups regarding normally distributed continuous variables. Wilcoxon rank test was used to test the difference between parity groups regarding non-normally distributed continuous data. Logistic regression was used to examine the association between parity groups and outcomes reporting relative risk (RR) with a 95% confidence interval (CI). Time-to-event curves were calculated by the Kaplan-Meier method to visualize the difference in hours to discharge between parity groups accompanied by a log-rank test. We adjusted for potential confounders selected a priori based on existing literature, supplemented by directed acyclic graphs for each outcome^22^. Missing data were reported in the tables. We performed an analysis of the selection process and loss to follow-up between one and six weeks after birth (Supplementary file Tables S4 and S5). Furthermore, we conducted a sensitivity analysis, assuming a best-case and a worst-case scenario (Supplementary file Tables S6 to S8). The data were analyzed using STATA/MP version 16.123. A p<0.05 was considered statistically significant in all analyses.

## RESULTS

**Figure 1:**
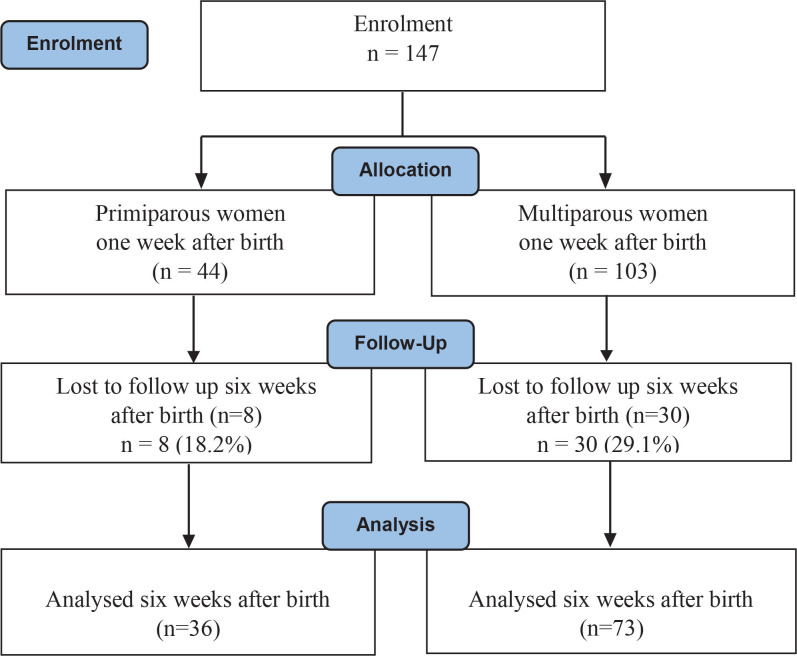
Flowchart of participation in the cohort study presented separately for primiparous and multiparous women

A total of 373 mothers met the inclusion criteria from 20 September 2020 to 16 April 2021. However, 226 women (60.6%) did not participate in the study, either because they did not receive an invitation, refused to participate, or did not respond to the questionnaire. A total of 147 (39.4%) answered the first questionnaire one week after birth, and 109 (74.1%) of these mothers answered the second questionnaire six weeks after birth (Figure 1). We observed no difference in the proportion of primiparous and multiparous women in the participating and non-participating group of mothers (Supplementary file Table S4). However, the participating women were healthier, and more mothers had uncomplicated births than the non-participating group. A smaller proportion of mothers in the participating group smoked during pregnancy (6.8% vs 9.3%). Furthermore, fewer mothers in the participating group had birth-related complications such as an induced birth, a vacuum-assisted birth, and bleeding more than 500 mL after birth. However, fewer had a long education in the participating group (9.3% vs 14.0%). Finally, more mothers in the participating group were discharged within six hours after birth. A total of 38 (25.9%) mothers were lost to follow-up at six weeks after birth (Supplementary file Table S5). In the group lost to follow-up, more mothers had a low level of education and reported less support at home. In addition, more mothers that were lost to follow-up were discharged within six hours after birth, and fewer mothers had oxytocin augmentation during birth compared to the mothers in the participating group (2.6% vs 12.2%). four hours longer before being discharged compared

The characteristics of mothers and newborns discharged within 24 hours after birth and differences between parity groups are presented in Table 1. Of the 147 participating mothers, 44 (29.9%) were primiparous women, and 103 (70.1%) were multiparous women. The newborns’ birth weight ranged between 2600 g and 5060 g. Significantly more primiparous women had epidural anesthesia as pain relief, oxytocin augmentation during birth, and a 3rd-degree or 4th-degree perineal tear than multiparous women. Further, more primiparous than multiparous women who were expected to leave the hospital within six hours after birth were discharged later than six hours after birth. On average, primiparous women stayed in the labor ward four hours longer before being discharged compared to multiparous women. Finally, more primiparous women reported having support from friends than multiparous women did. However, there was no difference in support from the father or co-parent or from family between parity groups.

**Figure 2:**
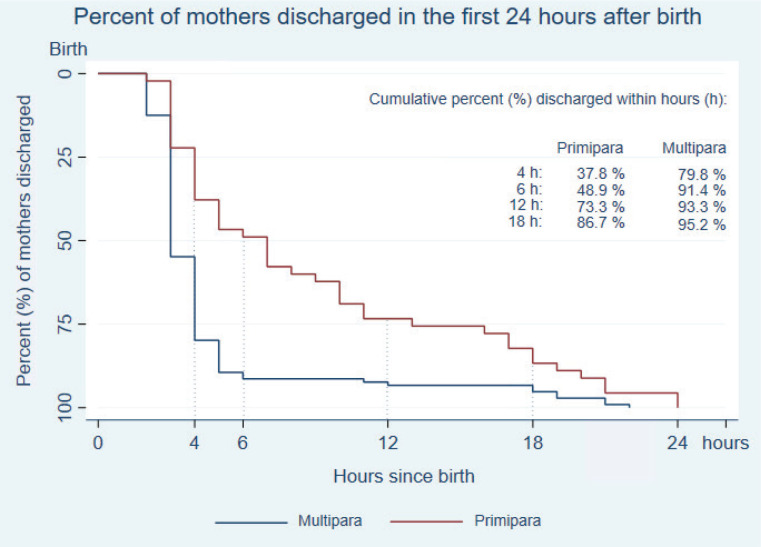
Kaplan-Meier curve illustrates the percentage of discharged mothers from birth at zero hour and with zero percent mothers discharged throughout the first 24 hours after birth. The curves are estimated separately for primiparous and multiparous women. The percentage of mothers discharged within 4, 6, 12 and 18 hours is presented separately for primiparous and multiparous women

Figure 2 illustrates the difference in hours from birth to discharge between primiparous and multiparous women. The curve shows that primiparous women stayed in the hospital longer than multiparous women throughout all 24 hours after birth. Over 90% of multiparous women left the hospital within six hours compared to 49% of primiparous women. Thus, there is a significant difference in the time for discharge between primiparous women and multiparous women (log-rank <0.001).

**Table 1:** Baseline characteristics of mothers and newborns discharged within 24 hours after birth according to primiparous and multiparous women (N=147)

*Characteristics*	*Total*	*Primiparas*	*Multiparas*	*p^a^*
	*n (%)^b^*	*n (%)^b^*	*n (%)^b^*	
**Total^c^**	147 (100.0)	44 (29.9)	103 (70.1)	
**Age** (years), median (range)	29 (20–41)	26 (20–35)	30 (21–41)	<0.001
<25	16 (10.9)	13 (29.5)	3 (2.9)	
25–29	76 (51.7)	28 (63.6)	48 (46.6)	
30–34	39 (26.5)	2 (4.5)	37 (35.9)	
≥35	16 (10.9)	1 (2.3)	15 (14.6)	
**Education level^c,d^**	129 (100.0)	38 (29.5)	91 (70.5)	
Skilled	25 (19.4)	5 (13.2)	20 (22.0)	≥0.05
Short	23 (17.8)	7 (18.4)	16 (17.6)	≥0.05
Medium	69 (53.5)	23 (60.5)	46 (50.5)	≥0.05
Long	12 (9.3)	3 (7.9)	9 (9.9)	≥0.05
**Caseload midwife in pregnancy** (yes)^c,d^	141 (100.0)	41 (29.1)	100 (70.9)	
In pregnancy	49 (34.8)	16 (39.0)	33 (33.0)	≥0.05
In pregnancy and birth	40 (28.4)	13 (31.7)	27 (27.0)	≥0.05
**Live alone** (yes)	5 (3.4)	1 (2.30)	4 (3.9)	≥0.05
**Social support**				
One or more sources (yes)^c,d^	128 (100.0)	37 (28.9)	91 (71.1)	≥0.05
From father/co-parent^e^	126 (98.4)	36 (97.3)	90 (98.9)	≥0.05
From family^e^	107 (83.6)	36 (97.3)	71 (78.0)	≥0.05
From friends^e^	61 (47.7)	26 (70.3)	35 (38.5)	<0.05
**BMI** (kg/m^2^), median (range)	26.4 (17–44)	26.3 (17–44)	26.4 (17–43)	≥0.05
<18.5	5 (3.4)	1 (2.3)	4 (3.9)	
18.5–24.99	76 (51.7)	26 (59.1)	50 (48.5)	
25.0–29.99	37 (25.2)	8 (18.2)	29 (28.2)	
30.0–34.99	10 (6.8)	2 (4.5)	8 (7.8)	
≥35	19 (12.9)	7 (15.9)	12 (11.7)	
**Smoking in pregnancy** (yes)	10 (6.8)	2 (4.5)	8 (7.8)	≥0.05
**Induction of labor** (yes)	36 (24.5)	11 (25.0)	25 (24.3)	≥0.05
**Epidural anesthesia** (yes)	23 (15.6)	13 (29.5)	10 (9.7)	<0.05
**Oxytocin augmentation** (yes)	18 (12.2)	10 (22.7)	8 (7.8)	<0.05
**Vacuum-assisted birth** (yes)	2 (1.4)	1 (2.3)	1 (1.0)	≥0.05
**3rd and 4th degree perineal tear** (yes)	3 (2.0)	3 (6.8)	0	<0.05
**Amount of bleeding** (mL)^c,d^	146 (100.0)	44 (30.1)	102 (69.9)	
≤500	137 (93.8)	40 (90.9)	97 (95.1)	≥0.05
501–1000	7 (4.8)	3 (6.8)	4 (3.9)	≥0.05
>1000	2 (1.4)	1 (2.3)	1 (1.0)	≥0.05
**Length of birth** (hours), median (range)	6.72 (0–36)	9.55 (1–32)	5.60 (0–36)	<0.001
≤12	128 (87.1)	32 (72.7)	96 (93.2)	
13–24	13 (8.8)	11 (25.0)	2 (1.9)	
>24	6 (4.1)	1 (2.3)	5 (4.9)	
**Gestational age** (weeks+days)				
37+0 – 37+6	6 (4.1)	3 (6.8)	3 (2.9)	≥0.05
38+0 – 38+6	13 (8.8)	6 (13.6)	7 (6.8)	≥0.05
39+0 – 39+6	37 (25.2)	8 (18.2)	29 (28.2)	≥0.05
40+0 – 40+6	47 (32.0)	9 (20.5)	38 (36.9)	<0.05
41+0 – 41+6	41 (27.9)	15 (34.1)	26 (25.2)	≥0.05
42+0 – 42+6	3 (2.0)	3 (6.8)	0	<0.05
**Birth weight** (g), mean ± SD	3665.8 ± 526.0	3601.0 ± 405.9	3693.3 ± 569.2	≥0.05
2500–3999	106 (72.1)	35 (79.5)	71 (68.9)	
≥4000	41 (27.9)	9 (20.5)	32 (31.1)	
**Neonatal suction after birth** (yes)	6 (4.1)	2 (4.5)	4 (3.9)	≥0.05
**Length of stay pp** (hours), median (range)	5.92 (2–24)	8.80 (2–24)	4.69 (2–22)	<0.001
≤6	116 (78.9)	22 (50.0)	94 (91.3)	
7–12	13 (8.8)	11 (25.0)	2 (1.9)	
13–24	18 (12.2)	11 (25.0)	7 (6.8)	
Expected discharged ≤6 hours pp^d^	24 (16.3)	18 (45.0)	6 (6.1)	<0.001

pp: postpartum. SD: standard deviation.

a p<0.05 statistically significant. Fischer’s exact test used on categorical data, Student’s t-test used on normally distributed data, Wilcoxon-rank test on non-normally distributed data.

b Column %.

c Row %.

d Missing values n (%): education 18 (12.2); caseload midwife 6 (4.1); social support 19 (12.9); amount of bleeding 1 (0.7); expected discharge ≤6 hours pp 8 (5.4).

e Multiple choices of social support were possible. A total of 100% is therefore exceeded when adding social support from father/co-parent, family, and friends. None reported no support at all.

Table 2 reports that a larger percentage of primiparous women reported adverse outcomes than multiparous women. Thus, the analysis documented that primiparous women had a 162% higher risk of having doubts about infant feeding after discharge than multiparous women. The percentage of primiparous women who did not breastfeed after six weeks increased from 2.3% one week after birth to 18.2%. In comparison, 12.3% of multiparous women did not breastfeed six weeks after birth. Furthermore, 54% of the primiparous women contacted the maternity ward after discharge, twice as many as multiparous women (54.1% vs 27.5%). Above 10% of the primiparous women reported no knowledge of their newborn’s well-being signs compared to 1% of multiparous women. There was an association between parity and knowledge of the newborn’s well-being signs in the crude analysis. However, the association was weak (95% CI: 1.39–96.35). The proportion of primiparous women who felt anxious or depressed was higher at one and at six weeks after birth compared to multiparous women. Finally, the proportion of newborns’ being readmitted was higher for newborns of primiparous women than newborns of multiparous women (9.1% vs 2.9%). Supplementary file Figure S1 illustrates the difference in proportions of primiparous and multiparous women who reported adverse outcomes after discharge.

**Table 2:** Relative risk (RR) for primiparous women compared to multiparous women discharged within 24 hours after birth regarding breastfeeding, doubts about infant feeding, contact with maternity ward, feelings of anxiety or depression, knowledge of signs of newborns well-being, and neonatal readmission (at one week N=147; at six weeks N=109)

	*Total n (%)*	*Yes n (%)*	*No n (%)*	*RR*	*95% CI*	*ARR^a^*	*95% CI*	*ARR^b^*	*95% CI*
**Breastfeeding at one week pp^c^**	143 (100.0)	139 (97.2)	4 (2.8)						
Primiparous women	44 (30.8)	43 (97.7)	1 (2.3)	0.75	0.08–7.01	0.38	0.04–4.06	0.56	0.04–8.00
Multiparous women (Ref.)	99 (69.2)	96 (97.0)	3 (3.0)	1		1		1	
**Breastfeeding at six weeks pp^c^**	98 (100.0)	84 (85.7)	14 (14.3)						
Primiparous women	33 (33.7)	27 (81.8)	6 (18.2)	1.48	0.59–3.90	1.03	0.36–2.92	1.18	0.42–3.31
Multiparous women (Ref.)	65 (66.3)	57 (87.7)	8 (12.3)	1		1		1	
**Doubts about infant feeding after discharge^c^**	137 (100.0)	33 (24.1)	104 (75.9)						
Primiparous women	45 (32.8)	19 (42.2)	26 (57.8)	2.81	1.56–5.06	2.57	1.33–5.00	2.62	1.35–5.10
Multiparous women (Ref.)	92 (67.2)	14 (15.2)	78 (84.8)	1		1		1	
**Contact with maternity ward <7 days after discharge^c^**	128 (100.0)	45 (35.2)	83 (64.8)						
Primiparous women	37 (28.9)	20 (54.1)	17 (45.9)	2.00	1.26–3.08	1.54	0.88–2.70	1.22	0.67–2.23
Multiparous women (Ref.)	91 (71.1)	25 (27.5)	66 (72.5)	1		1		1	
**Feeling anxious or depressed at one week pp**	147 (100.0)	10 (6.8)	137 (93.2)						
Primiparous women	44 (29.9)	5 (11.4)	39 (88.6)	2.34	0.71–7.68	2.52	0.62–10.20	2.75	0.67–11.19
Multiparous women (Ref.)	103 (70.1)	5 (4.9)	98 (95.1)	1		1		1	
**Feeling anxious or depressed at six weeks pp^c^**	104 (100.0)	6 (5.8)	98 (94.2)						
Primiparous women	34 (32.7)	3 (8.8)	31 (91.2)	2.06	0.44–9.67	0.92	0.18–4.45	1.33	0.18–10.05
Multiparous women (Ref.)	70 (67.3)	3 (4.3)	67 (95.7)	1		1		1	
**Knowledge of signs of newborns well-being after discharge^c^**	146 (100.0)	140 (95.9)	6 (4.1)						
Primiparous women	44 (30.1)	39 (88.6)	5 (11.4)	11.59	1.39–96.35	6.33	0.63–64.01	6.93	0.71–67.43
Multiparous women (Ref.)	102 (69.9)	101 (99.0)	1 (1.0)	1		1		1	
**Neonatal readmission <7 days after discharge**	147 (100.0)	7 (4.8)	140 (95.2)						
Primiparous women	44 (29.9)	4 (9.1)	40 (90.9)	3.12	0.73–13.36	2.14	0.32–14.12	1.70	0.17–17.00
Multiparous women (Ref.)	103 (70.1)	3 (2.9)	100 (97.1)	1		1		1	

pp: postpartum. ARR: adjusted relative risk.

a Adjusted for age and body mass index (BMI).

b Adjusted for age, body mass index (BMI), length of birth, and length of hospital stay after birth.

c Missing values n (%): breastfeeding at one week 4 (2.7); breastfeeding at six weeks 11 (10.1); doubts about infant feeding 10 (6.8); contact with maternity ward <7 days after discharge 19 (12.9); feeling anxious or depressed at six weeks pp 5 (4.6); knowledge of signs of the newborns well-being 1 (0.7).

Table 3 illustrates the mothers’ perception of the length of hospital stay after birth when discharged within 24 hours. Sub-analysis of mothers discharged before and after six hours were conducted. This study observed no association between parity groups and the perception of the hospital stay as too short. None of the mothers who stayed in the hospital more than six hours after birth found the time before discharge too short. However, 13.6% of primiparous women discharged within six hours after birth found the time too short compared to 5.4% of multiparous women. The sensitivity analysis assuming the best-case and worst-case scenarios included the 226 mothers who did not participate and 38 mothers who were lost to follow-up. The analysis did not find any association between parity of any of the outcomes, apart from doubts about infant feeding after discharge, thus indicating a robustness of the results (Supplementary file Tables S6 to S8).

**Table 3:** Primiparous and multiparous women’s perception of the length of hospital stay after birth when discharged within 24 hours after birth. Crude and adjusted relative risk (RR) of primiparous women versus multiparous women who perceived the length of hospital stay as too short versus appropriate or too long. The analyses were stratified by discharge time 0 to 6 hours after birth and 7 to 24 hours after birth (N=147)

	*Mothers’ perception of the length of hospital stay after birth*									
	*Total*	*Too short*	*Appropriate*	*Too long*	*Too short vs appropriate or too long*					
	*n (%)*	*n (%)*	*n (%)*	*n (%)*	*RR*	*95% CI*	*ARR^a^*	*95% CI*	*ARR^b^*	*95% CI*
**Discharged <24 hours after birth^c^**	146 (100)	8 (5.5)	136 (93.1)	2 (1.4)						
Primiparous women	44 (30.1)	3 (6.8)	41 (93.2)	0	1.35	0.35–5.60	1.43	0.31–6.52	1.42	0.31–6.55
Multiparous women (Ref.)	102 (69.9)	5 (4.9)	95 (93.1)	2 (2.0)	1		1		1	
**Discharged ≤6 hours after birth^c^**	115 (100)	8 (7.0)	106 (92.1)	1 (0.9)						
Primiparous women	22 (19.1)	3 (13.6)	19 (86.4)	0	2.54	0.66–9.82	2.26	0.54–9.41	2.25	0.54–9.42
Multiparous women (Ref.)	93 (80.9)	5 (5.4)	87 (93.5)	1 (1.1)	1		1		1	
**Discharged 7 to 24 hours after birth**	31 (100)	0	30 (96.8)	1 (3.2)						
Primiparous women	22 (71.0)	0	22 (100)	0	-		-		-	
Multiparous women	9 (29.0)	0	8 (88.9)	1 (11.1)	-		-		-	

ARR: adjusted relative risk.

a Adjusted for age and body mass index (BMI).

b Adjusted for age, body mass index (BMI), and length of birth.

c Missing values n (%): discharged <24 hours 1 (0.7); discharged ≤6 hours 1 (0.9).

## DISCUSSION

This study examined the association between parity and time for discharge, breastfeeding, and factors influencing breastfeeding. The study showed that primiparous women had a higher risk of having doubts about infant feeding after discharge than multiparous women. In addition, more primiparous women reported adverse outcomes than multiparous women. Finally, this study illustrated that primiparous women were consistently discharged later than multiparous women throughout the first 24 hours after birth. The consistency of each outcome will be discussed by comparing our results to the findings of other studies. We chose to divide the mothers into discharged within six hours and between seven and 24 hours. These cut-off values have been used in other studies, making our results comparable to those^2,3^.

### Parity and breastfeeding at one and at six weeks after birth

Only 2.8% of the mothers did not breastfeed one week after birth, indicating that most mothers had enough support to initiate breastfeeding. However, this study observed that more primiparous women discontinued breastfeeding six weeks after birth than multiparous women. This was an expected result since multiparous women with previous breastfeeding problems were offered hospitalization longer than 24 hours. Our findings correlate to a study, including 20694 mothers that documented primiparous women to have a shorter mean duration of breastfeeding than multiparous women^24^. However, the study did not report the time interval between giving birth and being discharged after birth. The study was conducted in the US, where the Newborns’ and Mothers’ Health protection Act ensures coverage for a 48-hour length of hospital stay after birth if the mothers have health insurance^25,26^. Therefore, we assume that most mothers in the study were discharged later than 24 hours after birth, thus making it difficult to compare the results with our findings. Some studies have compared parity and factors related to breastfeeding among mothers discharged within 24 hours after birth^2,3,27^. One study documented that maternal characteristic rather than the duration of hospital stay postpartum seemed to be essential predictors according to the duration of breastfeeding^2^. For example, low level of education and smoking were associated with a short breastfeeding duration^2^. On the other hand, the study showed that multiparity and a positive experience of initiating breastfeeding were associated with a long breastfeeding duration^2^. Nilsson et al.^3^ documented that breastfeeding experiences and breastfeeding knowledge were associated with discharge within 12 hours after birth. Therefore, parity, positive breastfeeding experience, breastfeeding knowledge, smoking, and a low educational level should be considered when developing policies for postpartum care.

### Parity and mothers feeling anxious or depressed at one and at six weeks after birth

This study did not find an association between parity and mothers feeling anxious or depressed one or six weeks after birth. However, twice as many primiparous women felt anxious or depressed than multiparous women at one and at six weeks after birth. The larger proportion observed in our study correlates with the findings of Nakamura et al.^13^ findings of more primiparous women being anxious and depressed than multiparous women within the first five days after birth measured on the Edinburgh Postnatal Depression Scale. We did not use a diagnostic tool to measure if the mothers were anxious or depressed in this study, but the results illustrate if the mothers felt anxious or depressed after birth. Furthermore, Nakamura et al.^13^ showed that the number of supporters at home is more strongly associated with maternal depression than mothers’ satisfaction with the support. Therefore, healthcare providers should consider the larger proportion of primiparous women feeling anxious or depressed when organizing the postpartum care after early discharge and consider the mother’s support at home when planning the follow-up strategy.

### Parity and doubts about infant feeding after discharge

An association between parity and the mothers’ doubts about infant feeding after discharge was documented in this study. Primiparous women’s risk of having doubts about infant feeding was 162% times as high as multiparous women’s risk. A randomized controlled trial confirmed that an educational program improved the parents’ knowledge of taking care of a newborn and improved the mothers’ self-efficacy^28^. In this study, parental education in pregnancy was converted into a 1-hour online video due to the national restrictions during the COVID-19 pandemic. This might have been reflected in our results and contributed to the doubts about infant feeding shown among the primiparous women.

### Parity and contact with the maternity ward within seven days after birth

More primiparous women than multiparous women contacted the maternity ward in the first seven days after discharge in this study. In total, 35% of the mothers contacted the maternity ward after discharge. Another study documented that 14% of mothers discharged within 12 hours called the maternity ward for advice after discharge^29^. The midwives offered these mothers as many home visits as needed^29^. In comparison, the mothers in this study were offered one home visit, which might explain the difference in the number of mothers contacting the maternity ward in the two studies. However, in this study, twice as many primiparous women contacted the maternity ward after discharge than multiparous women (54.1% vs 27.5%). The mother’s contact with the maternity ward can be interpreted as feeling insecure after early discharge and needing professional support. However, it also illustrates the will among primiparous women to ask for help from healthcare professionals when they have doubts about their or their newborn’s well-being, which might prevent neonatal and maternal morbidity.

### Parity and knowledge of signs of newborn’s well-being

There was no significant association in the adjusted analyses between parity and mothers having no knowledge about the signs of the newborn’s well-being. However, 11% of primiparous women reported not knowing the signs of the newborn’s well-being in this study, which indicates that primiparous women might need more information before discharge. Two studies documented that primiparous women wanted more information about caring for the baby, and multiparous women wanted more information about caring for themselves before discharge after birth^30,31^. However, the studies were from respectively 1987 and 1997. Thus, more updated research is needed to examine how primiparous and multiparous women differ in their need for information before early discharge after birth.

### Parity and the perception of the length of hospital stay after birth

No association between primiparous and multiparous women’s perception of the length of hospital stay was documented in this study. Only 5.5% of the mothers perceived the hospital stay too short when discharged within 24 hours after birth. In comparison, 13.5% found the hospital stay too short in a study from 2004 when mothers were discharged within 24 hours after birth^2^. We did not find any apparent reason for the difference in the proportion of mothers who perceived the length of hospital stay as too short. Thus, there is a lack of consistency in the literature regarding the mothers’ perception of the length of stay. However, twice as many primiparous women perceived the time before discharge after birth to be too short when discharged within six hours after birth compared to multiparous women. Furthermore, no mothers perceived the time before discharge too short when discharged seven to 24 hours after birth. The above mentioned indicate that some mothers might need more time before being ready for discharge, especially primiparous women. In this study, primiparous women were offered a home visit around 24 hours after discharge, which might impact their perception of the length of stay since they were asked at one week after birth. If a midwife offered all the mothers one or more home visits, fewer mothers might have perceived discharge within six hours as too short.

### Parity and the risk of neonatal readmission after early discharge

Neonatal readmission is a rare event, and a more extensive study population is needed to find any significant association between parity and the risk of neonatal readmission. Data from The Danish Health Data Authority show that in the Region of North Denmark, a total of 2.4% newborns of primiparous women and 2.9% newborns of multiparous women were readmitted within 30 days after discharge in 2018^32^. The larger number of readmitted newborns in this study than the average in the Region of North Denmark indicates that discharge within 24 hours might increase the risk for neonatal readmission. A large retrospective cohort study based on data from 1992–1995 in California included more than 1.2 million newborns delivered vaginally and examined the risk of newborns’ being readmitted when discharged on the day of birth^27^. The study documented that newborns of primiparous women had an increased odds ratio of 1.21 of being readmitted than newborns of multiparous women^27^. However, there was no description of the labor ward recommendation for the time of discharge, prenatal education, or the follow-up strategies after discharge in the study. Thus, more updated research is needed with a more detailed description of the setting.

### Strengths and limitations

There is a risk of selection bias caused by a loss of follow-up, and there is a risk of the results in the analyses at six weeks after birth being underestimated. In addition, this study did not have enough power to find a significant difference between the parity groups and breastfeeding at one and at six weeks after birth. This study is strengthened by using rigorous methods based on prespecified criteria in a protocol for cohort studies. Furthermore, the study was prospective, and there was only a minor risk of differentiated selection bias or information bias.

## CONCLUSIONS

The findings in this study indicate that differentiated postpartum care after early discharge might be needed, and special attention to primiparous women should be considered. Healthcare professionals should include the parents’ readiness for discharge before discharging mothers within six hours after birth to avoid mothers’ perceiving the time before discharge to be too short. Moreover, more individualized information about infant feeding and signs of the newborn’s well-being should be considered when developing policies for early discharge. Healthcare professionals might pay more attention to the mothers’ anxiousness or depression when developing postnatal care policies after early discharge. The results indicate that a follow-up strategy could be to allow the mothers to contact the maternity ward in the first seven days after discharge. The mothers’ doubts about infant feeding and the reasons for the mothers contacting the maternity ward after discharge need to be further examined to optimize postpartum care.

## Data Availability

The data supporting this research can be found in the supplementary content.
